# Microbial communities associated with thermogenic gas hydrate-bearing marine sediments in Qiongdongnan Basin, South China Sea

**DOI:** 10.3389/fmicb.2022.1032851

**Published:** 2022-10-25

**Authors:** Siwei Liu, Shan Yu, Xindi Lu, Hailin Yang, Yuanyuan Li, Xuemin Xu, Hailong Lu, Yunxin Fang

**Affiliations:** ^1^School of Earth and Space Sciences, Peking University, Beijing, China; ^2^Beijing International Center for Gas Hydrate, School of Earth and Space Sciences, Peking University, Beijing, China; ^3^National Research Center for Geoanalysis, Beijing, China; ^4^Guangzhou Marine Geological Survey, Guangzhou, China

**Keywords:** microbial diversity, thermogenic gas, natural gas hydrate, Illumina sequencing, marine sediments, South China Sea

## Abstract

Biogenic and thermogenic gas are two major contributors to gas hydrate formation. Methane hydrates from both origins may have critical impacts on the ecological properties of marine sediments. However, research on microbial diversity in thermogenic hydrate-containing sediments is limited. This study examined the prokaryotic diversity and distributions along a sediment core with a vertical distribution of thermogenic gas hydrates with different occurrences obtained from the Qiongdongnan Basin by Illumina sequencing of 16S rRNA genes as well as molecular and geochemical techniques. Here, we show that gas hydrate occurrence has substantial impacts on both microbial diversity and community composition. Compared to the hydrate-free zone, distinct microbiomes with significantly higher abundance and lower diversity were observed within the gas hydrate-containing layers. *Gammaproteobacteria* and Actinobacterota dominated the bacterial taxa in all collected samples, while archaeal communities shifted sharply along the vertical profile of sediment layers. A notable stratified distribution of anaerobic methanotrophs shaped by both geophysical and geochemical parameters was also determined. In addition, the hydrate-free zone hosted a large number of rare taxa that might perform a fermentative breakdown of proteins in the deep biosphere and probably respond to the hydrate formation.

## Introduction

Natural gas hydrates, mainly occurring in continental slope sediments under relatively high pressure and low temperature conditions, are crystalline solids containing low molecular gases in cage like structures constructed by water molecules, such as methane, ethane, CO_2_, etc. (Macdonald et al., 1994). Natural gas hydrates constitute an abundant form of carbon on Earth (Kvenvolden, 1993) and the most significant intermediate sink for the greenhouse gas methane, which appears to be dynamically stored in or released from hydrate reservoirs (Dickens, 2001b; Dickens, 2001a; Kvenvolden, 2002; Boetius and Wenzhöfer, 2013).

In nature, methane gas can be generated by two dominant processes, either “biogenic” or “thermogenic” (Whiticar, 1999). Biogenic methane is produced by microbial communities as a part of anaerobic respiration, whereas thermogenic gases are generated from thermocatalytic degradation of organic compounds at elevated temperatures (Schoell, 1988; Wuebbles and Hayhoe, 2002). The biogenic formation of methane is conducted by methanogenic archaea *via* three primary pathways: hydrogenotrophic, acetoclastic, and methylotrophic methanogenesis (Liu and Whitman, 2008; Thauer et al., 2008; Mayumi et al., 2016). In marine sediment, the majority of methane reservoir is consumed by microorganisms *via* the anaerobic oxidation of methane (AOM) mainly coupled with the reduction of sulfate, though iron, manganese, nitrate, and nitrite can also be used as electron acceptors in AOM processes (Barnes and Goldberg, 1976; Raghoebarsing et al., 2006; Beal et al., 2009; Ettwig et al., 2010; Yang et al., 2021). Typically, AOM happens in sulfate–methane transition zone (SMTZ) and is performed by microbial consortia where archaeal anaerobic methanotrophs (ANMEs) conduct reverse methanogenesis in association with bacterial partners (Barnes and Goldberg, 1976; Boetius et al., 2000; Reeburgh, 2007).

Relationships between the microbial structure and distribution and hydrated methane in deep marine sediments have received worldwide attention. Extensive studies have been conducted to characterize microbial diversity, activity and importance in methane hydrate-bearing subseafloor sediments, such as those in the Cascadia Margin (Cragg et al., 1995; Bidle et al., 1999; Lanoil et al., 2001, 2005; Marchesi et al., 2001; Mills et al., 2003, 2005; Knittel et al., 2005; Inagaki et al., 2006; Nunoura et al., 2008), Nankai Trough (Reed et al., 2002; Kormas et al., 2003; Newberry et al., 2004; Mills et al., 2012; Katayama et al., 2016, 2022), East Japan Sea (Yanagawa et al., 2014), Andaman Sea (Briggs et al., 2012), Ulleung Basin (Jeong et al., 2010; Lee et al., 2013; Ryu et al., 2013; Cho et al., 2017), Arctic (Carrier et al., 2020) and Shenhu area of the South China Sea (Liao et al., 2009; Lin et al., 2014; Jiao et al., 2015; Cui et al., 2019, 2020). A few of these studies were performed to compare the microbial communities in marine sediments with or without gas hydrate (Inagaki et al., 2006; Yanagawa et al., 2014; Jiao et al., 2015; Cui et al., 2020). Nevertheless, due to the wide distribution of biogenic hydrates, and the important role taken by microbes in methane metabolization, nearly all previous research had focused on the marine sediments containing biogenic methane, trying to figure out the microbiological processes that cycle the carbon in sediments rich in hydrates (Inagaki et al., 2006; Yanagawa et al., 2014; Jiao et al., 2015). It should not be neglected that the formation or dissociation of gas hydrate, even if they were not biogenic produced, could alter the geophysical and geochemical properties of the subseafloor sediments and further impact the microbial communities therein (Tomaru et al., 2004; Lee et al., 2008). In addition, heavier hydrocarbons (C_2+_) prevalent in the thermogenic hydrate-related gases might also influence the microbial distribution and metabolic potentials (Dong et al., 2019, 2020).

So far, it is still unclear the compositions and distributed characteristics of microbial communities inhabiting thermogenic gas hydrate-associated marine sediments, and whether they could also be distinguished from those found in the typical marine sediments without gas hydrates, as reported in the marine sedimentary environment with biogenic hydrates.

The Qiongdongnan Basin (QDNB) is one of the marine gas hydrate exploration targets in the South China Sea, where highly saturated gas hydrates were detected (Cui et al., 2019; Wei et al., 2019; Ye et al., 2019). Based on the carbon isotopic and field analysis of the composition of the hydrate-related gas, previous research has suggested that thermogenic gas might be the primary source of the hydrates in QDNB (Liang et al., 2019; Ye et al., 2019; Geng et al., 2021). This study aimed at characterizing the abundance and diversity of microorganisms in the marine sediments associated with thermogenic gas hydrates and determining spatial variations of the community structure along with hydrates distribution. We thus surveyed the microbial communities in a sediment core with the presence of multi-layer hydrates collected from the stable gas hydrate zone of QDNB, using molecular, geochemical techniques and statistical community analyses. We report that the microbial communities in thermogenic hydrate-bearing marine sediments of a QDNB core can be statistically distinguished from typical sediments with a significantly higher abundance and lower diversity, a remarkable stratified distribution of archaea along with various hydrate occurrences is also discovered. And it is speculated that a large population of rare taxa performing fermentative protein degradation metabolism was reduced by the gas hydrates formation.

## Materials and methods

### Site description and sample collection

**Figure 1:**
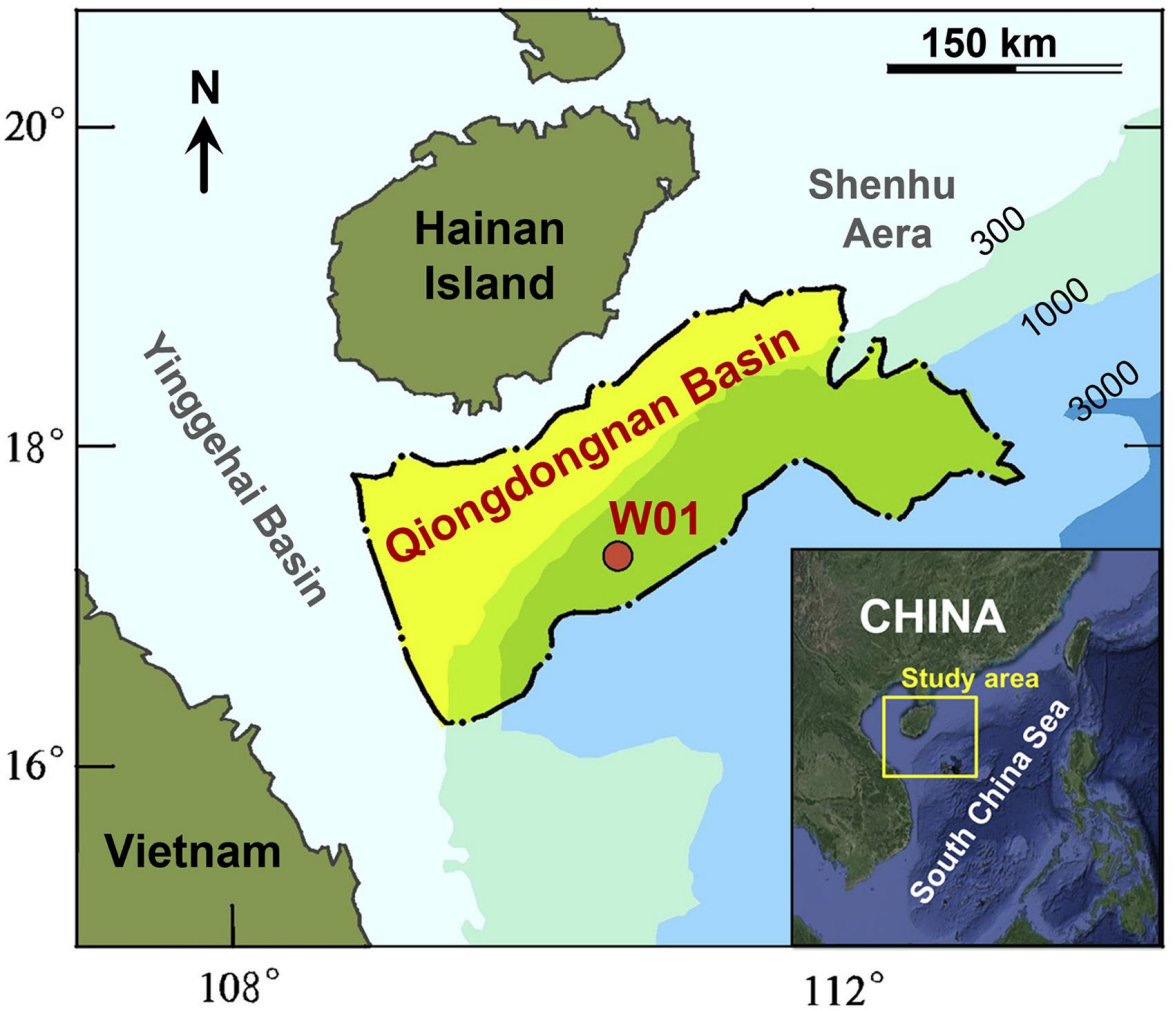
Location map of the Qiongdongnan Basin (QDNB), South China Sea. The red point refers to the coring site W01.

The study region is in the Qiongdongnan Basin, which is one of the potential gas hydrate-bearing basins on the northwestern continental slope of the South China Sea (Figure 1). The main components of the sediments are hemipelagic clayey silt and silty clay (Meng et al., 2021). Geophysical and geological characteristics related to fluid migration and gas hydrate deposits including mud diapirs, bottom simulating reflectors and gas chimneys are widespread in the area (Zhang et al., 2019). Previous studies had suggested that thermogenic gas might be the predominant source of hydrates at some sites in QDNB according to the carbon isotopic composition analyses (Huang et al., 2016; Liang et al., 2019; Lai et al., 2022; Ren et al., 2022), low C_1_/C_2+ (3–68)_ values of the hydrate-bound gases also indicated that thermogenic gas is the primary driver for the gas hydrate formation in QDNB (Ye et al., 2019).

Sediment core samples were obtained from the Qiongdongnan area at Site W01 which was drilled and cored to a depth of 176.7 m below seafloor (mbsf) in a water depth of 1513.89 m by pressure coring tools with Ball valve (PCTB) during the GMGS6 drilling expedition conducted in 2019 (Meng et al., 2021; Figure 1). Site W01 was positioned above deep subsurface gas chimneys, suggesting upstream gas fluxes within sediments (Meng et al., 2021). According to previous reports, the majority of natural gas in W01 is methane, supplemented by C2-C5 gases (Huang et al., 2016).

Detailed geochemical profiles which will be published elsewhere show that Core W01 exhibited significant gas hydrate indicators, and the pore-water sulfate was depleted throughout the core, indicating the sulfate–methane transition depth is located at 0 mbsf. Within the Core W01, subsamples used in this study were collected from three depth intervals: two gas hydrates bearing layers of 4.5–27.5 mbsf and 41–69 mbsf, respectively, and the 70–176 mbsf depth interval which was identified as a gas hydrate-free layer according to the drilling results. Based on the gas hydrate morphology classification method created by Holland et al., type II gas hydrates (fracture-filling gas hydrates exhibit vein, massive and nodular morphologies) were discovered in the 4.5–27.5 mbsf interval, while type I gas hydrates (disseminated pore-filling gas hydrate) were developed in the 41–69 mbsf (Holland et al., 2008; Meng et al., 2021). The occurrences of gas hydrates also seem to be affected by compositional changes in the hosting sediment, thus the hydrates of the studied layers could be further differentiated into three subtypes (II-b, I-b and I-a) according to lithological properties (Bahk et al., 2013).

In this study, a total of nine subsamples were retrieved from Core W01 using 50-mL cut-end syringes, including six samples with three hydrate types (I-a, I-b, and II-b) and three samples within the deep gas hydrate-free zone. The detailed sample information was summarized in Table 1. Subsamples were stored onboard at −20°C, transported on dry ice to the laboratory at the end of the expedition, and stored at −80°C until further processing.

**Table 1:** Information of the nine retrieved samples from Core W01 of QDNB.

Sample depth (mbsf)	Lithology	Gas hydrate types	
19	Silt	Fracture-filling	II-b
20	Silt	Fracture-filling	
42	Silt	Pore-filling	I-b
49	Silt	Pore-filling	
62	Fine sand	Pore-filling	I-a
64	Fine sand	Pore-filling	
71	Silt	Gas hydrate-free	N/A
73	Silt	Gas hydrate-free	
159	Silt	Gas hydrate-free	

N/A, not applicable.

### Geochemical analyses

The temperature was monitored by the inner T/P sensors during coring and processing. On board, pH measurements were made on pore water with a pH meter immediately after collection. Barium sulfate nephelometry and refractometry were used to quantify the pore water sulfate and salinity, respectively. The contents of anions (Cl^−^and Br^−^) were quantified using the standard method of ion chromatography (Metrohm 790 IC), with an analytical precision of 2%. The grain sizes of sediment samples were determined using a laser diffraction particle size analyzer (Mastersizer 3000, Malvern, United Kingdom) as described previously (Luo et al., 2012). The total organic carbon (TOC) of the sediments was measured by using a carbon/sulfur determinator (Series CS 744, Leco) in National Research Center for Geoanalysis, Beijing China. In detail, 200–300 mg samples were combusted at ~1,100°C inside the oven of the CS744 analyzer and the amount of carbon dioxide (CO_2_) generated was measured by an infrared cell. Samples were analyzed in duplicate to QA/QC for the homogeneity of the aliquots taken and analytical precision (Baux et al., 2019). Sediment samples were analyzed for major element compositions using X-Ray Fluorescence (XRF, GeniusIF, Xenemetrix Ltd.) with a precision of better than 8%. The δ^13^C and δ^18^O values of the bulk carbonate phase of subsamples were measured using a stable isotope ratio mass spectrometer (IRMS, Thermo Delta V) with an automated carbonate preparation device (GasBench II) as previously described (Liu et al., 2022).

### DNA extraction, PCR amplification, and Illumina MiSeq sequencing

For amplicon sequencing, microbial genomic DNA was extracted from 0.35 g of sediment using the FastDNA Spin Kit for Soil (MP Biomedicals) for polymerase chain reaction (PCR) amplification following the manufacturer’s instructions. For quantitative PCR (qPCR), microbial DNA was extracted and purified using the Natarajan et al. modified SDS-based method (Natarajan et al., 2016). The DNA concentrations were determined using a NanoDrop™ 2000 spectrophotometer (Thermo Fisher Scientific, United States).

The hypervariable V4 region of bacterial and archaeal 16S rRNA genes was amplified by PCR using a barcoded universal primer set Univ519F/Univ802R (Supplementary Table 2). PCR reaction mixtures contained 1 to 10 ng diluted DNA extracts, 1 × FastPfu buffer, 0.4 μmol/l of each primer, 250 μmol/l deoxynucleotides (dNTPs), 1 μl FastPfu polymerase (TransGen, China), and Mill-Q water added to a final volume of 50 μl. Thermal cycling was performed under the following conditions: initial denaturation at 95°C for 5 min, followed by 29 cycles of 94°C for 20 s, 50°C for 20 s, and 72°C for 25 s, and a final extension at 72°C for 10 min. A negative (no-template) control was used to exclude contamination. For each sample, amplicons from at least six independent PCR products were pooled and purified using EZNA Gel Extraction Kit (Omega Bio-Tek, United States) according to the manufacturer’s instructions and subjected to high-throughput sequencing using the Illumina MiSeq platform (Illumina Inc., United States) at Majorbio BioPharm Technology Co., Ltd., Shanghai China.

### Quantification of the bacterial, archaeal 16S rRNA genes and *mcrA* gene

To quantify the abundance of bacteria and archaea domain as well as anaerobic methanotrophs in the sediment samples, SYBR-Green-based qPCR was conducted with extracted DNA, using published specific primer sets (Supplementary Table 2). Each reaction (20 μl) contained 1× KOD SYBR qPCR Mix (TOYOBO, Co. Ltd), 5–8 ng template DNA, and 0.8 μM of each forward and reverse primer. All qPCR reactions were carried out using a CFX Connect Real-Time System (Bio-Rad, CA, United States).

Standard curves were generated using 10-fold dilutions of the known amount of purified PCR products of the target gene fragments. Each reaction was performed in triplicate. Melting curves were analyzed to detect the presence of primer dimers. The assay conditions are listed in Supplementary Table 2.

### Sequence processing

The 16S rRNA gene sequences were processed using Git for windows 2.28.0, R 4.2.0, Rstudio 1.4.1106, VSEARVH v2.15.2 (Edgar, 2010), and USEARCH v10.0.240 (Rognes et al., 2016). Specifically, raw sequence data were firstly checked by FastQC^1^ and processed as follows: joined paired-end reads and renamed by sample with the “-fastq_mergepairs” command, filtered low-quality reads (−fastx_filter command) after removing barcodes and primers by “-fastx_truncate,” removed redundant reads and singletons with the “-derep_fulllength” command. Thereafter, operational classification units (OTUs) were constructed using the “-cluster_otus” command at 97% sequence identity. OTUs were then mapped against the Silva 123 database (Quast et al., 2012) to remove sequences from chimera with the UCHIME algorithm in VSEARCH (−uchime_ref command). The taxonomy of the OTUs was classified with the Silva 123 database based on the sintax algorithm in VSEARCH (−sintax command) and the OTUs assigned to the chloroplasts and mitochondria were removed. Subsequently, the OTU table was obtained by USEARCH using the “fastx_getseqs” command.

### Bioinformatic and statistical analysis

For alpha and beta diversity, samples were rarefied at 37,320 sequences using R package vegan (Quast et al., 2012) corresponding to the lowest number of sequences in one sample. α-diversity indices, such as the specie richness, Chao 1, ACE, Shannon, Simpson, and Goods coverage, were calculated using the “-alpha_div” command of USEARCH, and the rarefaction curve was depicted by the package ggplot2. Distance matrices of Unifrac, Bray_Curtis, Jaccard, Manhatten and Euclidean were calculated with the “-beta_div” command, after constructing evolutionary trees based on OTUs. Heat maps of dissimilarity indices of the prokaryotic communities were depicted by GraphPad Prism 8.0.2, and the microbial communities’ dissimilarities among sediment groups were tested by ANOSIM.

Principal component analysis (PCoA) plot based on weighted Unifrac distances and Unweighted Pair Group Method with Arithmetic Means (UPGMAs) Clustering were displayed by vegan package, stats package and ggplot2 package in R software. Venn plots were constructed using R package ggvenn. Significantly different OTUs were visualized with a Volcano plot using the ggplot2 package. The heatmap of differentially abundant microbial taxa between hydrate-bearing and-free sediment groups was constructed by R package pheatmap based on the t-test (BH-adjusted *p* < 0.05) at the order level after scale normalization. The analysis of the process and scripts mainly referred to the study of Yongxin Liu (Liu et al., 2021).

The microbial function profiles of Core W01 microbiota were predicted using PICRUSt (phylogenetic investigation of communities by reconstruction of unobserved states, conducted on the ehbio online platform)^2^ to normalize the OTU table for 16S rRNA gene copy number variations, and then impute the functional microbial content of each sample based on KEGG orthologs (KO) and pathways. The heat map was then constructed using the R package devtools.

The effects of environmental variables on sediment microbial orders were judged using distance-based redundancy analysis (dbRDA) with the R package vegan. The Variance Inflation Factor Analysis (VIF) was performed prior to the RDA analysis in order to eliminate collinearity between environmental components and enhance efficiency. ANOVA-like permutation testing of constrained ordinations was performed using the R package statis. The plot was then visualized using the Canoco program for Windows 5.0.

The co-occurrence network was constructed to explore the associations between methanotrophs and other microbial taxa by calculating in R using the WGCNA package (Langfelder and Horvath, 2008). After the identification of the potential positive correlations between microbial families with Pearson’s *R* > 0.8 and BH-FDR-corrected *p* < 0.001, only families of methanotrophs and their associated taxa were retained for visualization using the interactive platform Gephi 0.9.5 with undirected network and the Fruchterman-Reingold layout (Bastian et al., 2009).

## Results

### Sediment geochemical conditions

**Figure 2:**
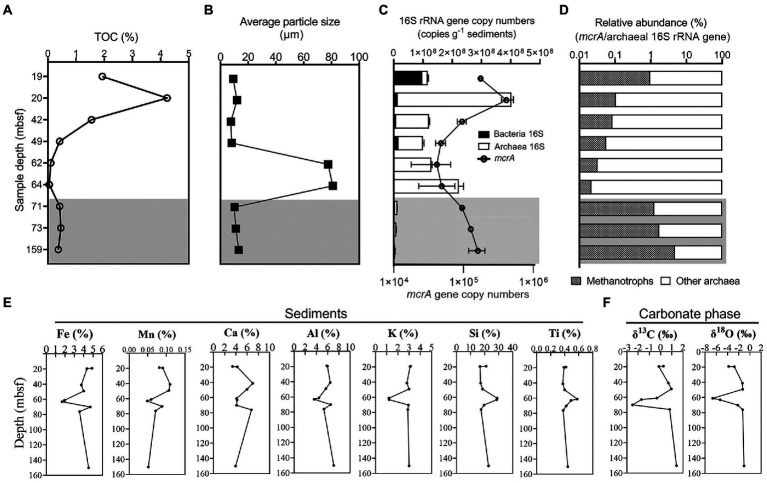
Depth profiles of TOC **(A)**, average particle size **(B)**, gene abundance of bacterial and archaeal 16S rRNA genes and *mcrA* **(C)**, the ratio of *mcrA* gene copies in archaeal 16S rRNA gene copies **(D)**, major elemental contents concentration **(E)** and stable carbon and oxygen isotopes composition of carbonate **(F)** of the sediment samples from Core W01. The gas hydrate-free zone is indicated by shaded area.

To understand the geochemical conditions where microbial communities occur, sediment samples from Core W01 were analyzed for total organic carbon (TOC), grain size, major elemental contents, stable carbon and oxygen isotopes composition of carbonate (δ^13^C_carb_ and δ^18^O_carb_). TOC values greater than 1% were detected in the top three sediment samples collected at 19, 20, 42 mbsf, while other deeper samples were with a lower TOC below 0.5% (Figure 2A). One sand layer was observed in Core W01 according to the results of particle size distribution: two sediment samples at 62 and 64 mbsf were fine sands with average grain sizes of 77.6 and 80.9 μm, respectively, while other sediment samples were composed of silt with smaller average particle sizes ranging from 7.43 to 13.18 μm (Table 1; Figure 2B; Supplementary Table 1). The lowest TOC content, chlorinity, Br concentration, salinity, and relative higher alkalinity were observed in these two samples (Supplementary Table 1), In addition, concentrations of Fe, Mn, Al, and K in the sandy samples at 62 and 64 mbsf were much lower while concentrations of Si and Ti exhibited higher values (Figure 2E), suggesting the geochemical anomaly of these two samples. Further, to confirm the origin of methane flux in the Site W01, we detected the carbon and oxygen isotope composition of carbonate in sediments which are affected by the percentage of AOM-driven carbonate. The results showed that the δ^13^C and δ^18^O values of carbonate in sediment (δ^13^C_carb_, δ^18^O_carb_) from Core W01 ranged from −2.50 to 1.42‰ and −6.64 to −1.05‰, respectively (Figure 2F; Supplementary Table 1), indicating a thermogenic origin of methane in the deep subsurface (Whiticar, 1999).

### Microbial abundance

Bacterial, archaeal 16S rRNA gene, and *mcrA* copy numbers were estimated by qPCR to represent the abundances of bacteria, archaea, and anaerobic methanotrophs, respectively. The results showed that bacterial and archaeal 16S rRNA gene copy numbers ranged from 1.14 × 10^6^ to 9.97 × 10^7^ and from 3.36 × 10^6^ to 3.88 × 10^8^ copies g^−1^ wet weight sediment, respectively (Figure 2C). The abundance of the archaeal 16S rRNA gene was generally one order of magnitude greater than that of bacteria but in one instance, the uppermost sample at 19 mbsf, where bacteria comprised 84% of the total prokaryotic community. Pearson’s analysis showed the total abundance of bacterial and archaeal 16S rRNA genes, which reached its maximum in the sample retrieved at 20 mbsf and decreased sharply in the three deepest hydrate-free samples at 71, 73, and 159 mbsf, was positively correlated to the TOC (*r* = 0.74, *p* = 0.024; Supplementary Figure 1) of the sediments.

The number of gene copies of *mcrA*, a methanotrophic archaea-specific gene, was detected in all collected sediment samples, varied between 4.16 × 10^4^ to 4.12 × 10^5^ copies g^−1^ wet weight sediment (Figure 2C). The proportion of methane metabolizing group in total archaeal cells of each sediment sample, estimated by the ratio of copy number of *mcrA* to archaeal 16S rRNA genes, decreased with depth in the upper hydrate-bearing sediment samples, from 0.96 and 0.02%, while raised in the three deep non-gas hydrate sediment samples retrieved below the gas hydrate zone, from 1.27 to 4.79% (Figure 2D).

### Alpha-diversity analysis

**Figure 3:**
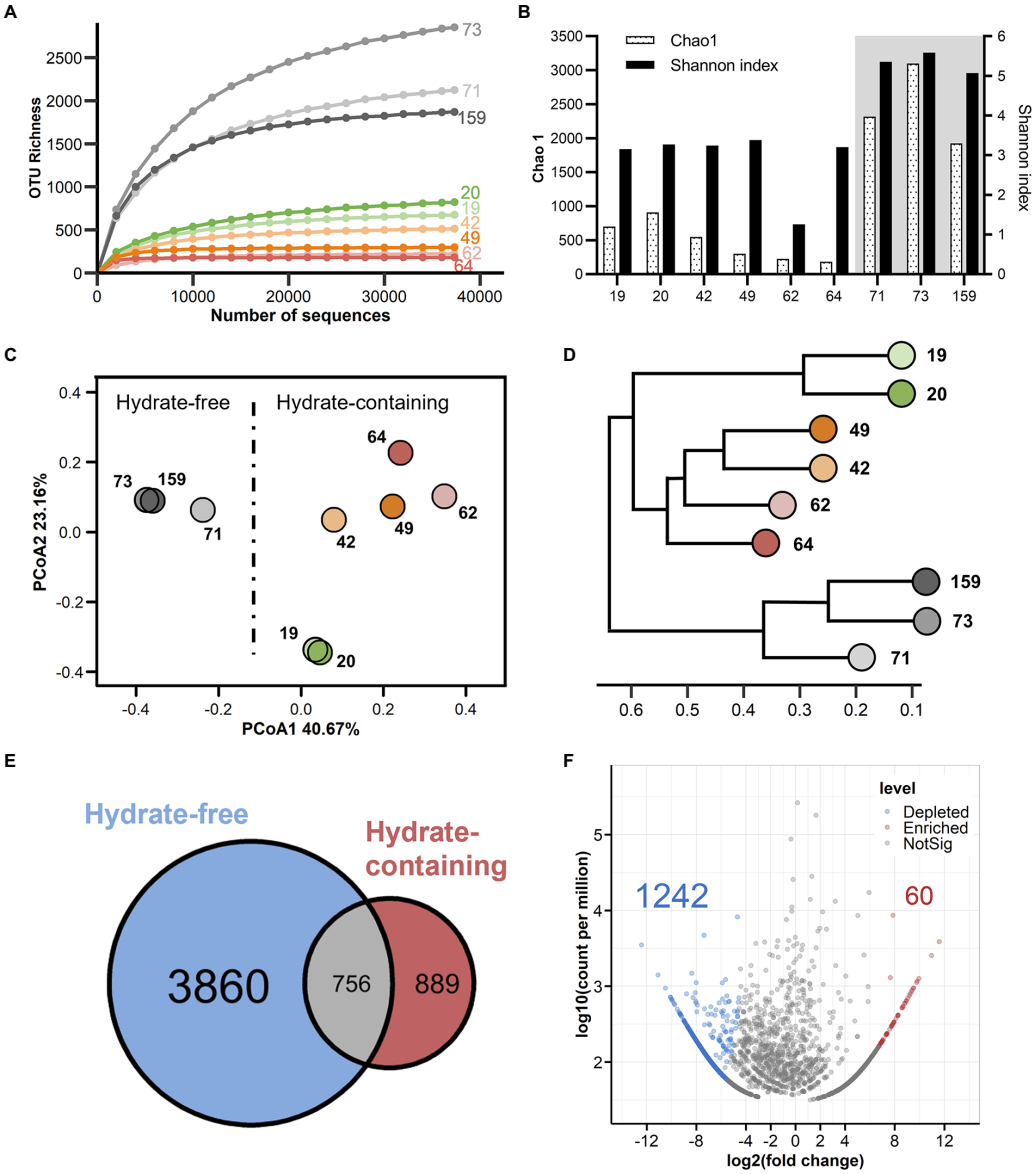
Alpha and beta diversity across W01 sediment column. **(A)** OTU-level rarefaction (observed species) curves. **(B)** Chao 1 estimated richness and Shannon index. The hydrate-free zone is indicated by shaded area. **(C)** Shift of microbial community structure in W01 sediment samples as visualized by Principal Co-ordinate Analysis (PCoA) based on the weighted Unifrac distance matrix. Each point represents the entire microbiological assemblage recovered from one sample; samples plotting closer to each other are more similar in microbial composition. **(D)** Dendrogram showing hierarchical clustering of the samples based on OTUs. **(E)** A Venn diagram displaying the degree of overlap of microbial OTUs between hydrate-free and hydrate-containing samples. **(F)** A Volcano plot illustrating OTUs that were significantly enriched (red) and depleted (blue) by hydrate-containing compared with hydrate-free samples as determined by differential abundance analysis. Enriched and depleted OTUs represent OTUs with two-fold higher or lower relative abundance (*p* < 0.05) in the hydrate-containing sediments, respectively.

Nine sediment samples covering the shallower hydrate-bearing layers to the deep hydrate-free zone of the Core W01 (for details, Table 1) were used for DNA isolation and prokaryotic diversity analysis. A total of 496,887 high-quality sequences were generated by MiSeq sequencing for all sediment samples (median = 59,094 sequences, ranging from 37,320 to 67,775 sequences). At a threshold of 97% sequence identity, 5,505 OTUs were identified (median = 674 OTUs, ranging from 182 to 3,088 OTUs, which comprises 5,388 bacterial OTUs and 117 archaeal OTUs, respectively (Supplementary Table 3). An overall dominance of bacterial sequences and high absolute numbers of bacteria OTUs were found throughout the core, accounting for 92.2–99.9% of the total sequence obtained from each sample. Species rarefaction curves all plateaued under the current sequencing depth (Figure 3A), suggesting that saturation in sequencing was achieved and those sequences covered all microbial species in the investigated W01 samples.

A vertical variation in microbial diversity was observed in W01. Gas hydrate-bearing sediments were characterized by relatively low Chao1 diversity reducing along with the depth from 19 to 64 mbsf, while surprisingly, Chao1 indexes steepened in the three samples collected from the deep hydrate-free zone, from 71 to 159 mbsf. The lowest values were calculated in the two sandy samples retrieved at 62 and 64 mbsf. Shannon indices revealed the same trends as the Chao1 estimator (Figure 3B; Supplementary Table 4). In addition, Chao 1, Shannon index, OTU Richness, Inverse Simpson index, and Ace index were all significantly increased in the three deepest samples, suggesting an apparent higher prokaryotic diversity in the gas hydrate-free zone of the Core W01 (Supplementary Table 5).

### Microbial composition and distribution

#### Bacterial community

**Figure 4:**
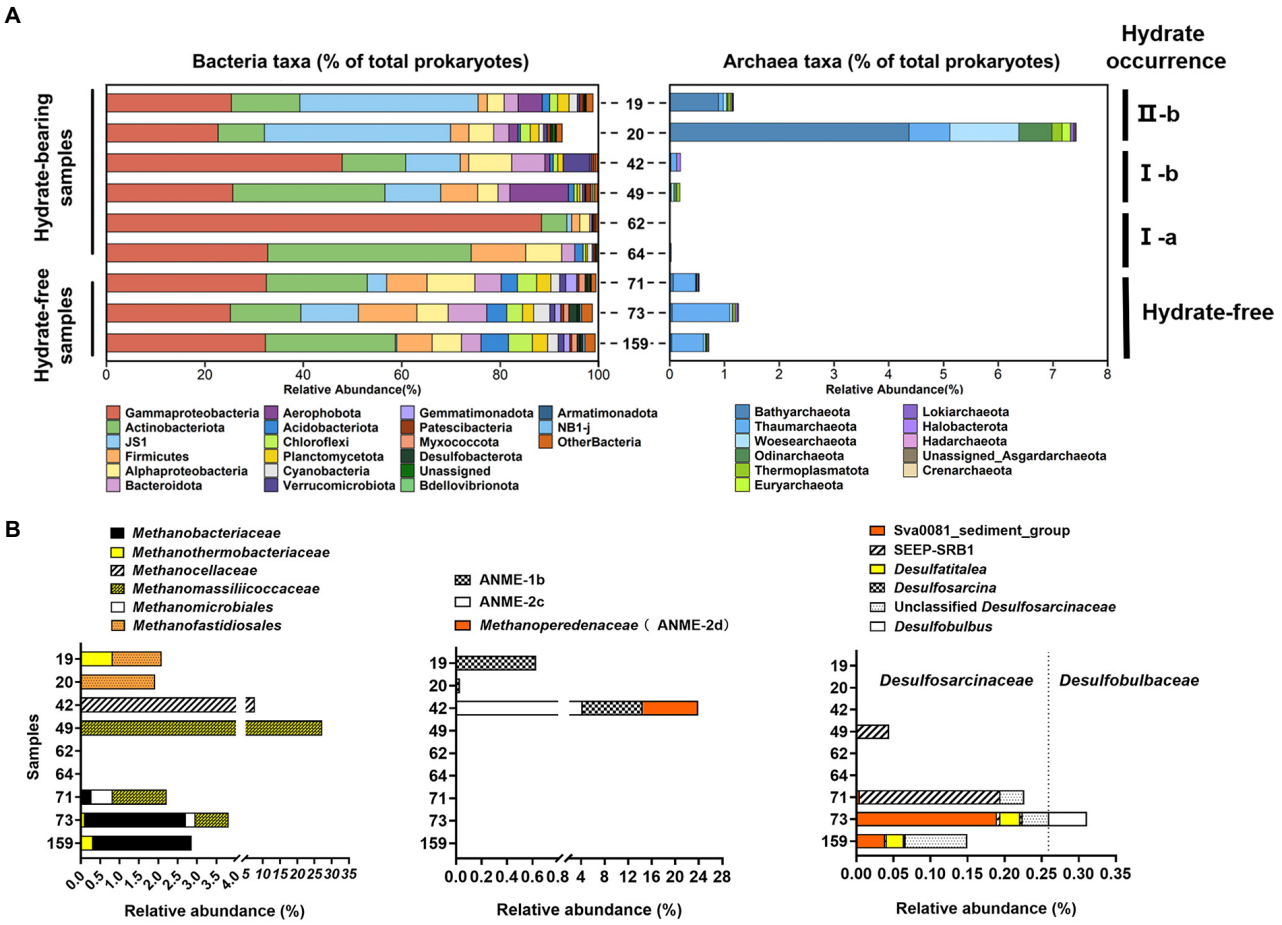
Microbial communities of nine sediment samples of Core W01 from QDNB based on Illumina MiSeq sequencing of 16S rRNA gene tagged amplicons. The percentage of the designated taxa within each sample is shown by colored bars. The distribution of **(A)** bacterial and archaeal communities are shown at the phylum level. Top 20 most abundant bacterial phylum and all archaeal phylum are listed. Relative abundance of sequences associated to **(B)** methanogen, ANME and deltaproteobacterial members of AOM consortia in sediments of Core W01. Percentages refer to the abundance of sequences of methanotrophs and sulfate reducers in total archaeal and bacterial sequences of each sample, respectively.

The microbial community structure was obtained based on the 16S rRNA genes V4 hypervariable regions with universal 16S primers targeting both bacteria and archaea. We revealed highly diverse bacterial communities with up to 76 phyla in W01 sediments. In general, the recovered sequences were dominated by Proteobacteria (with α-and γ-subdivisions), Actinobacterota, JS1 (Atribacteria), Firmicutes, and Bacteroidota at all depths, which comprised 85.12% of the total sequences. Compared to the hydrate-free samples, JS1 lineage and Aerophobota were generally detected with greater abundance in the silty gas hydrate-containing sediments, but not in sandy ones. Nevertheless, accounting for a few percent of the total, many taxa such as Acidobacteriota, Chloroflexi, Planctomycetota, Cyanobacteria, Gemmatimonadota and Myxococcota were more abundant in the hydrate-free zone (Figure 4A left panel). Additionally, sandy hydrate-associated samples from 62 and 64 mbsf were characterized by the lowest phylum varieties and dominated by fewer phyla, which is consistent with the results of the alpha-diversity analysis (Figure 3A left panel, Supplementary Table 4).

#### Archaeal community

In this study, a total of eleven archaeal phyla were found in the Core W01 samples, with the dominance of Bathyarchaeota, Thaumarchaeota, Woesearchaeota, Odinarchaeota, Thermoplasmatota, and Euryarchaeota accounting for 93.18% of total archaeal sequences. Remarkably, a sharp shift in archaeal composition along the vertical profile of the Core W01 was observed. The high relative abundance of archaea was found in the organic-rich sediment layers (19 and 20 mbsf) associated with II-b type gas hydrates and was dominated by Bathyarchaeota, accounting for 72.4 and 55.8% of the archaeal sequences in each sample, respectively. By contrast, archaeal groups were scarce within type I gas hydrated-containing sediments (from 42 to 64 mbsf). The lowest archaeal abundances (less than 0.3%) were detected in the sandy samples associated with I-a type gas hydrates from 62 to 64 mbsf, while archaeal communities in the deep hydrate-free zone (at 71, 73, and 159 mbsf) with moderate sequence abundance around 1% were dominated by Thaumarchaeota, accounting for an average relative abundance of 78.98% in the archaeal community of each sample (Figure 4A right panel).

#### Anaerobic methanotrophs

A remarkable niche partitioning of the methanotrophic community along with the Core W01 was found. Sequences affiliated with methanogens and ANMEs represented 3.08 and 0.60% of the total archaeal sequences for W01 sediments, respectively. Except for the two sandy samples at 62 and 64 mbsf where methanotroph-related sequences were not recovered, methanogen-affiliated sequences were detected throughout the sampling depth with relative abundances from 2 to 30% in archaea. Methanogens in samples collected at 19 and 20 mbsf were dominated by *Methanofastidiosales*, accounting for 1.83% of archaeal reads in these two samples. Samples at 42 and 49 mbsf where the relative abundances of methanogens reached their peak, hosted *Methanocellaceae* and *Methanomassiliicoccaceae* respectively, accounting for 10 and 30% of the total archaeal sequences in each sample, respectively. *Methanobacteriaceae* and *Methanomicrobiales* were only found in the deep hydrate-free zone as dominant clades, while *Methanothermobacteriaceae* and *Methanomassiliicoccaceae* were found in both hydrate-containing and-free layers (Figure 4B left panel).

ANME group within W01 sediments was only detected in the top three sediment samples containing hydrates, samples at 19 and 20 mbsf were composed solely of ANME-1b, accounting for 0.66 and 0.03% of archaeal reads of each sample, respectively. Sample 42 contained the highest abundance of the ANME group with 24.35% of archaeal reads, comprised of ANME-1b, ANME-2c, and *Methanoperedenaceae* (ANME-2d; Figure 4B middle panel). However, δ-proteobacterial sulfate-reducing bacteria were only found in the hydrate-free zone with low abundance (Figure 4B right panel), indicating no niche overlap between ANMEs and their potential sulfate-reducing partner, we speculated that some sulfate reduction processes in these sediments were coupled to the oxidative breakdown of organic matter.

### Beta-diversity analysis

The variation in the community structure of the W01 sediment samples was analyzed by Principal Co-ordinates Analysis (PCoA) and hierarchical clustering analysis. The first two PCoA axes explained a total of 63.8% of the variation. Sediment samples retrieved from the hydrate-bearing and hydrate-free zones separated along axis 1 on the PCoA plot based on the weighted Unifrac distance (Figure 3C). Also, hydrate-bearing and-free sediment samples clustered separately on the UPGMA tree based on the Bray-Curtis analysis. These results indicated that hydrate-containing and-free sediments contained distinct microbial communities (Figure 3D). Notably, sediment-associated microbial assemblages also demonstrated clear differentiation among the four studied layers (II-b, I-b, I-a and hydrate-free layers) of Core W01 with various hydrate occurrences according to ANOSIM tests with limited samples (Supplementary Figure 2E).

In the set of taxa of this study, the prokaryotic communities were composed of very few abundant taxa and a great many rare ones, with the top three most abundant OTUs comprising 45.61% of all sequences (data not shown). Venn diagrams were used to display the common and unique OTU numbers to intuitively describe the similarity and overlap between sample groups. Our results showed that a high number of unique OTUs were detected in the hydrate-free sediment samples, accounting for 69.70% of the total OTUs and 10.03% of the total sequences (Figure 3E; Supplementary Figure 2D). Furthermore, differentially OTUs between these two groups were further visualized in a Volcano plot. In detail, compared to the hydrate-free sediments, the hydrate-bearing samples showed 60 enhanced OTUs (1.09%) and 1,242 depleted OTUs (22.56%). Among the depleted OTUs, 1,237 OTUs (86.67%) were rare taxa (abundances below 0.1%), indicating that the rare community in the hydrate-free samples made major contributions to the significant difference between the two groups (Figure 3F). This conclusion was further confirmed by the analysis of the microbial order with significant differences (*p* < 0.05), except for uncultured *Thermoleophilia*, 71 of the total 72 differentially abundant orders in hydrate-bearing sediments were depleted (Supplementary Figure 3). These 72 orders accounted for 9.85% of the total sequences, with an average abundance of each order of only 0.14%. Together, these differences indicated that the formation of gas hydrates in Core W01 might modulate the taxonomic microbial groups by reducing the number and abundance of deep marine rare taxa.

**Figure 5:**
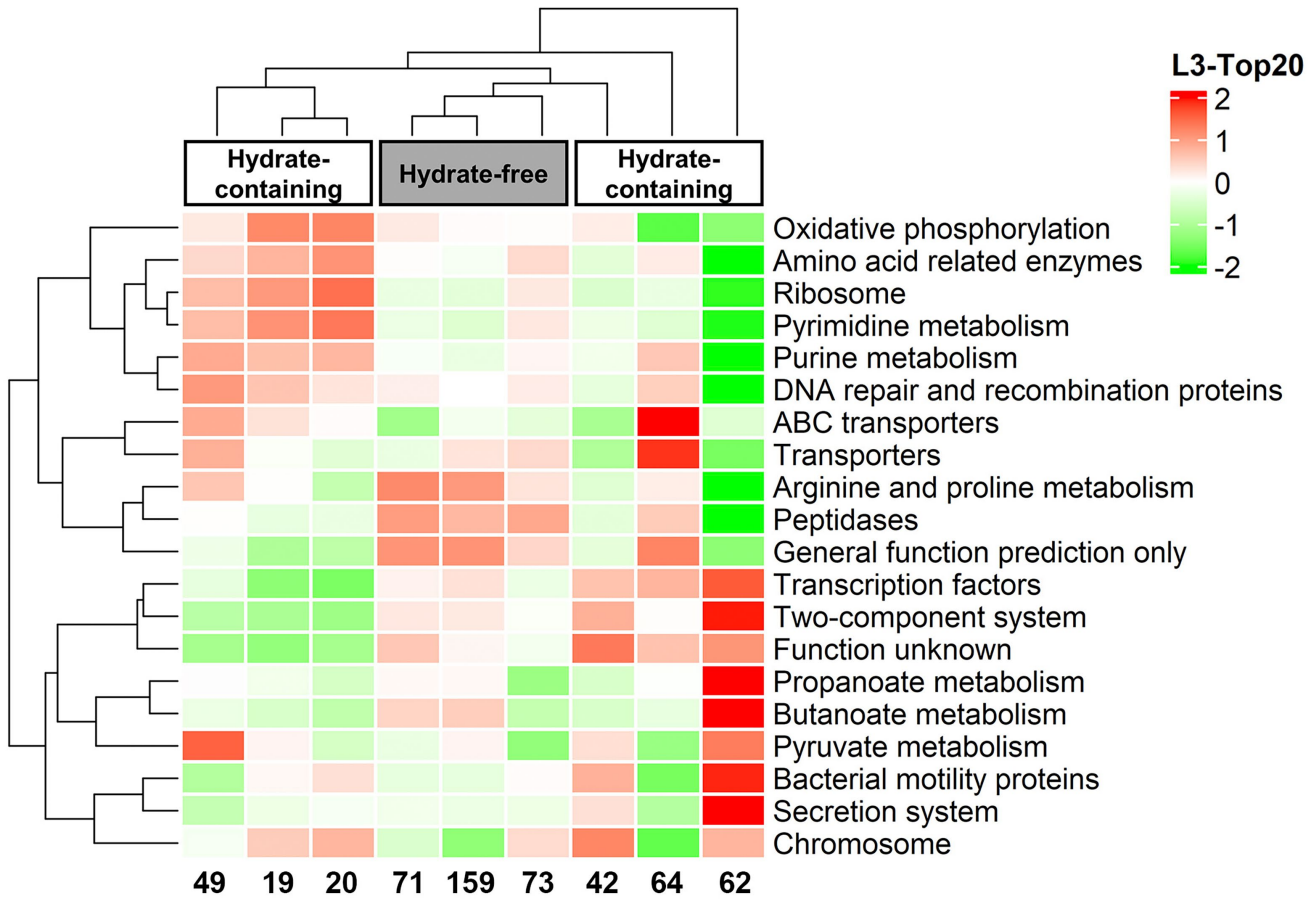
Heatmap profile of the top 20 most abundant KEGG (level-3) metabolic pathways created by metabolic function prediction associated to the microbiome profiles.

Further, the sequence data were employed in PICRUSt analysis to predict the functional diversity of the microorganisms in each sample. A heatmap was made from the top 20 KEGG orthologue (KO) groups according to functional annotation and abundances, and all groups were clustered based on functional relative abundance. As seen from the heatmap, the functional potential of microbial communities from Core W01 differed along the depth, in particular, gas hydrate-free samples had a higher KO abundance in protein metabolic pathways such as “Arginine and proline metabolism” and “Peptidases” (Figure 5).

### Environmental drivers shaping the distribution of microbial taxa

**Figure 6:**
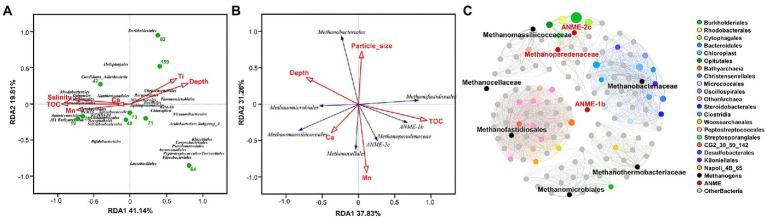
The impact of different environmental parameters on microbial community and the co-occurrence network analysis. Distance-based redundancy analysis (dbRDA) plot based on environmental variables fitted to the taxonomic composition of the microbial taxa **(A)** and anaerobic methanotrophs **(B)** at the order level. The green dots represent 9 detected sediment samples in the QDNB sediments; environmental parameters and microbial taxa are represented as red arrows and blue arrows, respectively. **(C)** Co-occurrence patterns of the microbial families regarding the relationship of the methanotrophic archaea with other taxa in Core W01. Edges joining the nodes signify significantly strong positive correlations (*p* < 0.001, *r* > 0.8). Nodes are colored by corresponding orders assigned to different lineages, and the node size reflects the relative abundance of each microbial family.

To determine the effect of sediment properties on microbial community composition, the property variables were analyzed using Distance-based Redundancy Analysis (dbRDA), where depth, TOC and concentrations of Ti were the most contributing factors as environmental input (Figure 6A). The first dbRDA axis correlating with all variables of the sediment explained 41.14% of the total variation and separated the top from bottom sediment samples, and the second axis of dbRDA explained 19.81% of the fitted variation. dbRDA analysis revealed that the abundance of JS1 lineage, *Oceanospirillales* and *Bathyarchaeia* were related to TOC and concentrations of Mn, and the abundance of *Christensenellales*, *Thermomicrobiales*, and *Bacteroidales* had positive correlations with depth and Ti concentrations (Figure 6A).

We further identified which set of variables better explains variations in the relative abundance of ANME and methanogen groups by using dbRDA. In this case, 70.37% of the correlation between methane-metabolizing archaeal composition and environmental data was explained by two axes, demonstrating the reliability of the results. A strong niche preference was shown with TOC and Mn concentrations being the main predictor variables (Figure 6B). ANME groups are all located in the bottom right quadrant of the dbRDA plot, showing strong correlations with Mn concentration and TOC. *Methanobacteriales* showed the highest positive correlation with the average particle size, *Methanofastidiosales* were associated with high levels of TOC, *Methanocellales* showed strong positive correlations with Mn concentrations, while *Methanomicrobiales* and *Methanomassiliicoccales* correlated strongly with depth (Figure 6B).

The co-occurrence network analysis showed that methanogenic and ANME families formed individual clusters with other corresponding taxa and there was no shared node (Figure 6C), further confirming the niche separation of the methane-metabolizing archaea in the Core W01. In particular, ANME families were significantly and positively correlated with highly abundant bacterial orders *Burkholderiales*, *Rhodobacterales* and *Cytophagales*; *Methanofastidiosales* were significantly and positively correlated mainly with *Bathyarchaeia* and low abundant families in both bacterial and archaeal domain; *Methanobacteriaceae* were significantly and positively correlated with *Bacteroidales*, *Chloroplast*, *Kiloniellales* and *Oscillopirales* (Figure 6C). The ecological interactions between methanogens/ANMEs and other microbial taxa are in line with the fact that the methanotrophs and some heterotrophic microbial species in deep-sea sediments could interact metabolically *via* an anaerobic food chain.

## Discussion

In marine sediments where hydrates are present, a considerable proportion of the methane is thermogenic. In addition to QDNB, gas hydrates containing thermogenic hydrocarbons have been discovered in a variety of locations, including the Gulf of Mexico (Sassen and MacDonald, 1994; Sassen et al., 2004), the Caspian Sea (Diaconescu et al., 2001), the Black Sea (Woodside et al., 2003; Mazzini et al., 2004), and the Japan Sea (Matsumoto et al., 2005). Thermogenic gas hydrates are more likely to be found in deep sediments about 1,000–2,000 mbsf (or even deeper) in most regions and much below the methane hydrate stability zone (Lu et al., 2007). It is anticipated that more thermogenic complex gas hydrates will be discovered when deeper wells are drilled. The relationships between microbial distributions, cell abundance, and sediment geochemistry as well as physical characteristics in thermogenic hydrate systems have not yet been fully investigated. Our study characterized microbial biogeography with respect to this environmental system using sediment core samples from the QDNB area.

### High relative abundance of archaea in Core W01

In this study, qPCR results showed that the abundance of 16S rRNA gene of archaea is generally ten times more than that of bacteria except for in the shallowest sample at 19 mbsf, however, our sequencing results with a universal primer set showed an overwhelming dominance of bacterial sequences. It has been methodologically challenging to accurately quantify the cell numbers of a certain microbial group in marine environments (Morono et al., 2014), and there is no universally acknowledged method for quantifying bacteria and archaea in marine sediments, and different techniques have yielded highly conflicting results with identical samples (Lloyd et al., 2013a). According to Lipp et al., which is consistent with our qPCR results, archaea are more abundant than bacteria in marine systems (Lipp et al., 2008), previous studies on marine hydrate-rich environments might underestimate archaeal biomass due to the limitation of detection techniques (Lipp et al., 2008; Nunoura et al., 2008; Ye et al., 2009). We speculate that the inconsistency between the two methods was caused by the PCR primers discrepancy or the incomplete archaeal cell lysis using the DNA extraction kit. Although the primer pair we adopted for the amplicon sequencing was reported to have superior predicted coverage of the taxonomic diversity of archaea and bacteria (Klindworth et al., 2012; Starnawski et al., 2017), it appears to be possible that our primer set is a poor match to the 16S rRNA gene sequences of most archaea in the Core W01. In future studies, sequence amplification with Archaea-specific primers might be helpful to produce the most informative profiles of archaea and to further confirm our observation in the QDNB area. Commercial DNA extraction kits are thought to produce better comparable results, especially among different individuals due to the process standardization (Natarajan et al., 2016), but archaeal cells from the W01 sediment samples might not be completely lysed because certain archaeal cells are resistant to lytic protocols of the Kit that are effective for bacterial cells, due to unusual cell wall structures (Roopnarain et al., 2017). Our results indicated that the handcrafted SDS-Based DNA extraction method we used for the qPCR template preparation might recover a larger amount of the genomic DNA of archaea with better performance of cell lysis efficiency. Archaea are predicted to be better adapted in extreme low-energy settings like those present in the deep biosphere, while bacteria thrive in more dynamic environments (Valentine, 2007). However, due to the longer life cycles and community turnover times in the deep subsurface, archaea with higher abundance do not necessarily govern the biogeochemical processes (Lipp et al., 2008).

### Potential microbial indicators for marine hydrate systems

The association between the microbial community composition and the occurrence of gas hydrate and depth has been established earlier in hydrate-containing marine sediments (Lanoil et al., 2001; Inagaki et al., 2006). In the Core W01, similarly, of the present study, microbial community structure between the thermogenic hydrate-containing and-free sediments was significantly different. For gas hydrate exploration, microbial taxa that can serve as complementary indicators have long been looked for. In the Core W01, notably, the JS1 lineage and *Aerophobetes* (Aerophobota phylum) were found to be more abundant in silty hydrate-containing sediment compared to hydrate-free samples, but not in sandy ones where they presented at very low abundance. JS1 is a prevalent bacterial group in subseafloor sediments and has been frequently found to be dominant in some strictly anoxic organic-rich sediments with gas hydrates or significantly correlated to the hydrate presence, indicating a hydrate-containing habitat preference (Reed et al., 2002; Inagaki et al., 2006; Nunoura et al., 2008; Jeong et al., 2010; Lee et al., 2013; Ryu et al., 2013; Yanagawa et al., 2014; Katayama et al., 2016; Cho et al., 2017). A recent genomic study suggested that JS1 has the potential for fermentation using a variety of substrates and for syntrophic acetate oxidation coupled with hydrogen or formate scavenging methanogens (Lee et al., 2018). Aerophobota is a newly defined bacterial phylum that is widely distributed in deep-sea sediments (Chaumeil et al., 2019). Although some previous research has shown evidence of saccharolytic and fermentative metabolism in at least some members of *Aerophobetes*, the diversity and metabolic capacities of this taxon are still poorly understood (Wang et al., 2007; Rinke et al., 2013; Wang et al., 2016). *Aerophobetes* have been detected in the gas hydrate-bearing zone of sediment cores in relative higher abundance compared to hydrate-free sediments in the South China Sea earlier (Cui et al., 2019, 2020) and in some cold seep environments (Wang et al., 2016; Huang et al., 2019). It has been discovered that gas hydrate dynamics could be affected by properties of gas hydrate-bearing sediments, such as particle size, sediment components and porosity (Lu et al., 2011; Kumari et al., 2021). At depths of 62 and 64 mbsf, elements Fe, Mn, Ca, Al, and K in sediments showed sharp decreases, while Si and Ti increased. The anomaly of these major element content suggests a significant difference in paleoclimate and mineralogical composition in these two sandy samples, which might mainly comprise quartz or silicate (Wei et al., 2003; von Eynatten et al., 2016). It is possible that microbial activities may also associate with the change of these elements. The dbRDA showed a significant impact of Mn, Ca and Ti content on certain microbial groups, though the reason for this is not clear, we speculated elements with electrovalence change such as manganese content in the sediments might reflect a redox change in the local environment (Ingram et al., 2016).

It should be noted that the correlation between hydrate presence and these two bacterial lineages with hydrate-containing habitat preference was only detected in the silty samples, suggesting the limitations of the microbial index while applied to sediments of different lithology.

Further, our results showed that in the Core W01 the archaeal communities, including anaerobic methanotrophic groups shifted sharply along with discrete layers, while the bacterial changes along these layers were rather moderate. Remarkably, sandy hydrate-bearing samples at 62 and 64 mbsf showed the lowest microbial diversity, and only four archaeal OTUs were found in this layer with low abundances (data not shown). This vertically stratified distribution of microbial communities in the subseafloor has been reported in several studies, emphasizing the significance of environmental characteristics, including the physical and chemical factors, on microbial populations (Reed et al., 2006; Wilms et al., 2006; Li et al., 2008; Pachiadaki et al., 2011). Particularly, Mills et al. showed a significant difference of archaea communities between the sediment-entrained hydrate and interior hydrate in Gulf of Mexico hydrate ecosystems whereas bacteria were not distinguishable, indicating that archaea might be less tolerant to environmental fluctuations and more specialized for particular environmental circumstances (Mills et al., 2005). The observed congruence of archaeal community and vertical changes with gas hydrate-bearing dynamics in our study suggested that archaeal communities might respond to the occurrence of gas hydrates and are more sensitive to lithology and hydrate morphologies. Microorganisms, especially anaerobic methanotrophs exhibited distinct distributions according to geochemical variables previously unidentified. Thus, the copresence of archaeal members related to hydrate-bearing marine sediments could be further investigated to look for the microbiological indicators for the potential locales, accumulation, and morphologies of the thermogenic hydrate in deep marine sediments.

### Methanotrophs in Core W01

In sediment samples of Core W01, methanogens only exist in a small fraction of marine sediments where a considerable amount of methane is present, indicating the trivial contribution of the methanogenesis process. A total of five orders of methanogen were found, accounting for approximately 0.03% of prokaryotes. Among them, *Methanobacteriales*, *Methanomicrobiales*, and *Methanocellales* are hydrogenotrophs, which are able to reduce CO_2_ using H_2_ as an electron donor to produce methane (Liu and Whitman, 2008). *Methanofastidiosa* and *Methanomassiliicoccales*, two newly described novel methanogens, were reported to perform methanogenesis by an H_2_–dependent methylotrophic pathway, according to recent genomic and transcriptomic evidence (Zhang et al., 2020). Thus, our results indicated that hydrogenotrophic and methylotrophic methanogenesis is the dominant pathways for biogenic methane production in Core W01. In addition, the ANMEs group, including ANME-1b, ANME-2c, and *Methanoperedenaceae* (ANME-2d) were detected in the uppermost three samples where no 16S rRNA gene fragments of sulfate-reducing deltaproteobacterial partners were found. ANME-1b which was the only subgroup found in all these three samples constituted a major component of the ANME cluster, accounting for 50% of the ANME sequences. As mentioned in previous studies, ANME-1 could be independent of bacterial association, our findings support the above observation. Based primarily on the observations that ANME-1 was more prevalent in the sulfate-depleted methanogenic zone of sediments, several studies have suggested that ANMEs, and ANME-1 in particular, may be capable of producing methane, primarily (Lloyd et al., 2011; Yanagawa et al., 2011; Jagersma et al., 2012; Bertram et al., 2013). However, Yoshinaga et al. (2014) pointed out that due to the carbon back flux from AOM, SMTZ may extend into the methanogenic zone where ANME may actually carry out AOM under sulfate-depleted conditions (Yoshinaga et al., 2014). We failed to amplify *mcrA* genes to use as markers for anaerobic methanotrophs despite many attempts with different primer sets reported by previous studies. We speculated the reason could be the measly percentage of genomic DNA from methanotrophs in the extracted PCR templates, it is also possible that the organization of the *mcrA* in methanotrophs from the W01 site is slightly different than in other counterparts.

### Important role played by the rare taxa

Despite the fact that microbial communities commonly contain a large number of rare taxa that constitute the majority of the observed membership, it is unknown what role this microbial “rare biosphere” plays in the dynamics of the community. Previous studies suggested that most environmental metabarcoding studies are not sequencing deep enough and OTUs/taxa represented by low-abundance sequences such as singletons, doubletons, and tripletons may be valuable and informative in highlighting rare lineages in communities (Zhan et al., 2014). Rare microbial taxa may contribute to nutrient cycling in great diversity, serving as a reservoir that can rapidly respond to environmental changes and foster community stability in a variety of settings (Shade et al., 2014). In this study, a unique structural perspective of the subseafloor microbial populations was produced by the highly resolved sequence dataset obtained at an average sequencing depth of ten million reads per sample. Our results showed numerous microbial OTUs (5,436 out of 5,505 total OTUs) that individually represent less than 0.1% of the sequence highlighting the community’s overall diversity. Surprisingly, a prominent high microbial diversity comprised of a large number of rare taxa was observed in the deep hydrate-free sediments with TOC of less than 1%, indicating the presence of gas hydrate in marine sediments could have a selection effect on the microbial communities.

Below the methanogenesis zone, the food chain begins with fermentation and is rather brief (Parkes et al., 2000). According to Lomstein et al. (2012), most of the available organic materials in deep sediments are microbial necromass. Because cells are primarily composed of proteins by weight, most of the fermentable necromass is in the form of proteins and amino acids (Lomstein et al., 2012). It has been suggested that protein degradation seems to be a crucial metabolism for some subsurface microorganisms (Lloyd et al., 2013b) and peptidases were more active than other enzymes in similar settings (Coolen and Overmann, 2000; Jacobson Meyers et al., 2014). Our results of predictive functional profiles of microbial communities in W01 sediment samples showed that peptidases synthesis, as well as arginine and proline metabolism, were significantly more abundant in the deep hydrate-free sediment samples in contrast to hydrate-containing sediments, indicating fermentative breakdown of proteins is a crucial strategy to support deep biospheres and could be inhibited by the gas hydrate formation.

## Conclusion

In this study, high-throughput sequencing of the prokaryotic 16S rRNA genes combined with molecular and geochemical analyses was applied to investigate the microbial characteristics in the Core W01 of QDNB with multi-layer thermogenic hydrates. Based on our results, the following conclusions have been reached:

1. In the Core W01, the abundance of archaea cells was ten times higher than that of bacteria.
2. Distinct microbial abundance and diversity related to the thermogenic gas hydrate presence were identified.
3. The bacterial taxa of all samples are dominated by *Gammaproteobacteria* and Actinobacteria. The phyla ‘Atribacteria’ lineage JS1 and Aerophobota were significantly abundant in the silty hydrate-containing sediments, other than in sandy ones; while the archaeal communities changed dramatically throughout the vertical profile of sediment layers and a spatial heterogenicity of anaerobic methanotrophs was also determined.
4. The deep hydrate-free sediments harbored a large number of distinct rare microbial groups presumed to perform protein degradation and might respond to the hydrate formation.
5. The distribution of microbial taxa is related to the hydrate existence, occurrences and environmental parameters including sedimentary facies.

Our research offers a novel perspective on the distributions of microbial species associated with thermogenic gas hydrates and adds new insights to the ecological role of the methane reservoir on Earth.

## Data availability statement

The datasets presented in this study can be found in online repositories. The names of the repository/repositories and accession number(s) can be found at: http://gsa.big.ac.cn/index.jsp, PRJCA011124.

## Author contributions

SY designed the study, conducted the experiment, helped with analysis, and wrote the manuscript in consultation with all other authors. SL performed the phylogenetic and statistical analyses and revised this manuscript. XL performed the experiments for nucleic acid extractions and quantitative PCR and revised this manuscript. HY, YL, and XX did the geochemical experiments. YF collected and shipped the samples and provided porewater parameters. HL revised this manuscript. All authors contributed to the article and approved the submitted version.

## Funding

This work was financially supported by China Geological Survey (grant number DD20221703).

## Conflict of interest

The authors declare that the research was conducted in the absence of any commercial or financial relationships that could be construed as a potential conflict of interest.

## Publisher’s note

All claims expressed in this article are solely those of the authors and do not necessarily represent those of their affiliated organizations, or those of the publisher, the editors and the reviewers. Any product that may be evaluated in this article, or claim that may be made by its manufacturer, is not guaranteed or endorsed by the publisher.

## Footnotes

^1^ http://www.bioinformatics.babraham.ac.uk/projects/fastqc/

^2^ http://www.ehbio.com/ImageGP/index.php/Home/Index/PiCrust.html
