# Supplementary material for: Microbial communities associated with thermogenic gas hydrate-bearing marine sediments in Qiongdongnan Basin, South China Sea
Source: Front Microbiol. 2022 Oct 25;13:1032851. doi: 10.3389/fmicb.2022.1032851 (PMC9640435; doi:10.3389/fmicb.2022.1032851)
Supplement: Supplementary file 4 [file Table_1.DOCX]

**Supplementary Table 1.** Summary of *in situ* temperature, experimental results of pore water chemistry, TOC and the average particle size of the W01 sediment samples from QDNB.

| Samples | Temperature  (℃) | Cl^−^, corrected  (mM) | Br^−^, corrected  (mM) | Salinity, corrected (mM) | pH | TOC  (%) | Average particle size (μm) |
| --- | --- | --- | --- | --- | --- | --- | --- |
| 19 | 5.5 | 945.62 | 1.48 | 53.96 | 8.06 | 1.93 | 9.08 |
| 20 | 5.5 | 806.83 | 1.26 | - | 8.17 | 4.23 | 11.91 |
| 42 | 7.3 | 699.87 | 1.16 | 38.54 | - | 1.55 | 7.43 |
| 49 | 7.8 | 624.45 | 1.03 | 36.47 | - | 0.42 | 8.06 |
| 62 | 8.8 | 229.46 | 0.33 | 15.28 | 8.69 | 0.1 | 77.60 |
| 64 | 9.0 | 179.86 | 0.25 | 10.81 | 9.08 | 0.05 | 80.90 |
| 71 | 9.6 | 552.65 | 0.96 | - | - | 0.42 | 9.99 |
| 73 | 9.8 | 544.54 | 0.94 | 33.55 | - | 0.46 | 11.00 |
| 159 | 16.4 | 590.45 | 1.07 | 35.42 | 8.12 | 0.37 | 13.18 |

‘-’, not determined.
