# Supplementary material for: Microbial communities associated with thermogenic gas hydrate-bearing marine sediments in Qiongdongnan Basin, South China Sea
Source: Front Microbiol. 2022 Oct 25;13:1032851. doi: 10.3389/fmicb.2022.1032851 (PMC9640435; doi:10.3389/fmicb.2022.1032851)
Supplement: Supplementary file 5 [file Table_2.DOCX]

**Supplementary Table 2.** List of primers and assay conditions used in PCR and qPCR efficiencies.

| Primer | Target DNA | Predicted target group | Sequence (5'-3') | Annealing temp (℃) | Average efficiency | R^2^ | Reference |
| --- | --- | --- | --- | --- | --- | --- | --- |
| Univ519F | 16S (V4) | Bacteria and archaea | CAGCMGCCGCGGTAATWC | 51.3 | N/A | N/A | (Starnawski et al., 2017) |
| Univ802R | 16S (V4) | Bacteria and archaea | TACNVGGGTATCTAATCC | 51.3 |  |  | (Starnawski et al., 2017) |
| Bac908F | 16S (qPCR) | Bacteria | AACTCAAAKGAATTGACGGG | 60.5 | 99.67% | 0.998 | (Starnawski et al., 2017) |
| Bac1075R | 16S (qPCR) | Bacteria | CACGAGCTGACGACARCC | 60.5 |  |  | (Starnawski et al., 2017) |
| Arch915F | 16S (qPCR) | Archaea | AATTGGCGGGGGAGCAC | 65.5 | 99.54% | 0.993 | (Starnawski et al., 2017) |
| Arch1059R | 16S (qPCR) | Archaea | GCCATGCACCWCCTCT | 65.5 |  |  | (Starnawski et al., 2017) |
| mlas | *mcrA*  (qPCR) | Methanogens/ANME | GGTGGTGTMGGDTTCACMCARTA | 55 | 101.32% | 0.996 | (Steinberg and Regan, 2008) |
| mcrA-rev | *mcrA*  (qPCR) | Methanogens/ANME | CGTTCATBGCGTAGTTVGGRTAGT | 55 |  |  | (Steinberg and Regan, 2008) |

N/A，not applicable.

**Reference**

Starnawski, P., Bataillon, T., Ettema, T.J.G., Jochum, L.M., Schreiber, L., Chen, X., et al. (2017). Microbial community assembly and evolution in subseafloor sediment. *Proceedings of the National Academy of Sciences* 114(11)**,** 2940-2945. doi: 10.1073/pnas.1614190114.

Steinberg, L.M., and Regan, J.M. (2008). Phylogenetic Comparison of the Methanogenic Communities from an Acidic, Oligotrophic Fen and an Anaerobic Digester Treating Municipal Wastewater Sludge. *Applied and Environmental Microbiology* 74(21)**,** 6663-6671. doi: 10.1128/aem.00553-08.
