# Supplementary material for: Microbial communities associated with thermogenic gas hydrate-bearing marine sediments in Qiongdongnan Basin, South China Sea
Source: Front Microbiol. 2022 Oct 25;13:1032851. doi: 10.3389/fmicb.2022.1032851 (PMC9640435; doi:10.3389/fmicb.2022.1032851)
Supplement: Supplementary file 6 [file Table_3.DOCX]

**Supplementary Table 3.** Sequence data of the nine subsamples of Core W01. Paired reads after quality filtering assigned to Bacteria and Archaea are 491567 and 5320 sequences, respectively.

| Samples | Number of reads (n) | Number of OTUs (n) | OTUs assigned to Bacteria (n) | OTUs assigned to Archaea (n) |
| --- | --- | --- | --- | --- |
| 19 | 37 320 | 674 | 638 | 36 |
| 20 | 40 368 | 836 | 780 | 56 |
| 42 | 59 094 | 540 | 530 | 10 |
| 49 | 67 763 | 298 | 293 | 5 |
| 62 | 67 775 | 230 | 227 | 3 |
| 64 | 45 910 | 182 | 181 | 1 |
| 71 | 67 038 | 2 271 | 2 243 | 28 |
| 74 | 67 555 | 3 088 | 3 052 | 36 |
| 159 | 44 064 | 1 888 | 1 869 | 19 |
