# Supplementary material for: Microbial communities associated with thermogenic gas hydrate-bearing marine sediments in Qiongdongnan Basin, South China Sea
Source: Front Microbiol. 2022 Oct 25;13:1032851. doi: 10.3389/fmicb.2022.1032851 (PMC9640435; doi:10.3389/fmicb.2022.1032851)
Supplement: Supplementary file 7 [file Table_4.DOCX]

**Supplementary Table 4.** Alpha diversity indices calculated for each sample.

| Samples | Species richness | Chao1 | Ace | Shannon | Simpson | Goods coverage |
| --- | --- | --- | --- | --- | --- | --- |
| 19 | 677 | 710.52 | 717.47 | 3.16 | 0.17 | 0.997747 |
| 20 | 829 | 901.04 | 923.19 | 3.28 | 0.17 | 0.996332 |
| 42 | 552 | 575.02 | 572.79 | 3.25 | 0.10 | 0.999187 |
| 49 | 303 | 310.20 | 308.13 | 3.39 | 0.09 | 0.999867 |
| 62 | 227 | 230.27 | 230.12 | 1.27 | 0.63 | 0.999867 |
| 64 | 184 | 189.00 | 187.61 | 3.24 | 0.11 | 0.999892 |
| 71 | 2249 | 2313.24 | 2318.05 | 5.38 | 0.05 | 0.997381 |
| 73 | 3034 | 3078.84 | 3101.01 | 5.60 | 0.04 | 0.997201 |
| 159 | 1876 | 1903.81 | 1907.84 | 5.08 | 0.08 | 0.997723 |
