# Supplementary material for: Microbial communities associated with thermogenic gas hydrate-bearing marine sediments in Qiongdongnan Basin, South China Sea
Source: Front Microbiol. 2022 Oct 25;13:1032851. doi: 10.3389/fmicb.2022.1032851 (PMC9640435; doi:10.3389/fmicb.2022.1032851)
Supplement: Supplementary file 8 [file Table_5.DOCX]

**Supplementary Table 5.** Analysis of alpha-diversity between Gas hydrate-bearing sediment samples and gas hydrate-free sediment samples of Core W01 by paired T-test.

| Index | Hydrate-bearing sediments | Hydrate-free sediments |
| --- | --- | --- |
| Shannon 2.92 | 2.92±0.82* | 5.34±0.26 |
| Inverse-Simpson | 7.27±3.63* | 20.21±6.40 |
| OTU Richness | 451.70±256.90** | 2280.00±509.90 |
| Chao 1 | 478.00±290.30** | 2446.00±345.10 |
| Ace | 482.10±292.70** | 2459.00±620.60 |
| Good’s coverage | 0.99±0.001 | 0.99±0.004 |

**p*<0.01 vs. gas hydrate-free sediments, ***p*<0.001vs. hydrate-free sediments.
